# Emergence and spread of *Hyalomma* ticks and Crimean-Congo haemorrhagic fever in Europe: a systematic review

**DOI:** 10.1186/s13071-025-07104-3

**Published:** 2025-10-28

**Authors:** Mohammed Masud Parvage, Jerome N. Baron, Jan C. Semenza, Anna Omazic

**Affiliations:** 1 Department of Chemistry, Environment and Feed Hygiene, Swedish Veterinary Agency (SVA), 75189 Uppsala, Sweden; 2 Heidelberg University Hospital (UKHD), 69120 Heidelberg, Germany; 3 Department of Epidemiology and Global Health, Umeå University, 90187 Umeå, Sweden

**Keywords:** Ticks, Tick-borne diseases, Vector-borne diseases, Zoonoses, Public health, One Health, Surveillance, Early warning systems

## Abstract

**Background:**

Crimean-Congo haemorrhagic fever (CCHF) is a severe, often fatal zoonotic disease caused by the CCHF virus (CCHFV). It is primarily transmitted by *Hyalomma* ticks, which serve as both reservoir and vector. While these ticks are endemic to Asia, Africa, and parts of Europe, recent detections in previously unaffected European regions raise concerns about the potential spread of the disease.

**Objectives:**

This study aims to systematically review the first detections of *Hyalomma* ticks, CCHFV, and CCHF disease in Europe. The goal is to support surveillance strategies and enhance preparedness for future outbreaks.

**Methods:**

A systematic literature search was conducted across six international databases, complemented by targeted searches. From 1315 articles retrieved (1235 from systematic and 80 from targeted searches), 92 were included based on relevance to the presence of *Hyalomma* ticks, CCHFV, and/or CCHF in Europe.

**Results:**

*Hyalomma* ticks are either endemic or have been detected at least once in 40 European countries. CCHFV and CCHF diseases have been reported in 24 and 22 countries, respectively. The distribution of *Hyalomma* ticks has expanded because of environmental and climatic changes, as well as increased movement of birds, livestock, and humans. CCHF has been present in Europe for decades, with early reports from Bulgaria, Russia, Ukraine, and Kosovo. More recent detections include Georgia, Spain, the UK, and Portugal.

**Conclusions:**

The ecological and climatic suitability for *Hyalomma* ticks is increasing across Europe, heightening the risk of CCHFV transmission. Integrated entomological surveillance and virological monitoring are essential for early detection and rapid response to potential outbreaks. Strengthening these efforts will be critical to containing the spread of CCHF and protecting public health.

**Graphical Abstract:**

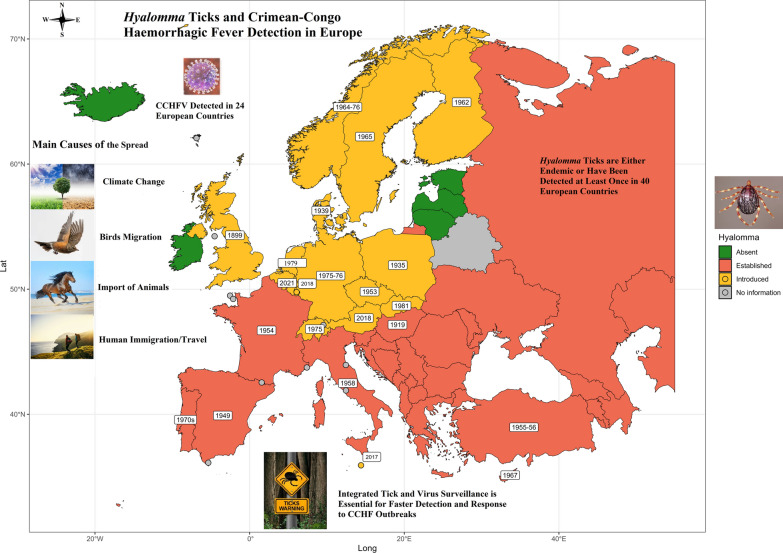

**Supplementary Information:**

The online version contains supplementary material available at 10.1186/s13071-025-07104-3.

## Background

Globally, ticks are the second most important arthropod disease vector, after mosquitoes, and transmit pathogens to both humans and animals [1]. Due to ongoing environmental and climatic changes, the threat level of zoonotic tick-borne disease transmission in Europe has increased [2], notably from ticks of the Ixodidae family [3–6]. Ticks of the genus *Hyalomma* are of both medical and veterinary concern, as they can transmit pathogens to humans and domestic animals, primarily infesting cattle and horses, thereby inflicting medical and economic harm [7–10]. In humans, the health impacts from these pathogens can range from asymptomatic to severe and even result in death, with a case fatality rate of up to 40% [11–13]. The global economic burden associated with the management of tick-borne diseases in veterinary medicine is estimated to be between 22 and 30 billion USD annually [9].

Among tick-borne zoonoses in Europe, Crimean-Congo haemorrhagic fever (CCHF) is of particular concern due to the severity of the disease and its high case fatality rate. It is the most widespread tick-borne viral haemorrhagic disease and is caused by an arbovirus called Crimean-Congo haemorrhagic fever virus (CCHFV). This belongs to the genus *Orthonairovirus* (family Nairoviridae, order Bunyavirales). CCHFV is a biosafety level 4 pathogen (i.e. it has a high risk of aerosol transmission and can cause severe to lethal infections). The World Health Organisation has designated the CCHF disease as one of the seven highest-priority epidemic-prone diseases due to its epidemic potential, high fatality rates (3–40%), and lack of effective treatment measures [7, 11, 14–17]. CCHFV is mainly transmitted to humans in two ways: (1) through the bite or by direct contact with secretions or fluids of an infected tick and (2) by direct contact with secretions, fluids, blood, tissue, or organs from an infected host during the critical phase of infection [7, 18]. Vertical transmission [19–21] and nosocomial outbreaks in the sanitary environment, including those related to aerosol generation at a hospital's isolation facilities and accidental cases in the laboratory when handling viral material, have also been reported [22–24]. The virus can survive throughout the tick’s life cycle [25]. People at high risk of CCHFV infections include people working in the agro-pastoral or animal husbandry fields, in contact with fresh meat and blood from animals (i.e. slaughterhouse personnel), and health care providers [11].

Ticks of the genus *Hyalomma* serve as both reservoirs and vectors of CCHFV. While their natural distribution is primarily confined to parts of Asia, Africa, and southern Europe, recent surveillance has documented their presence in several additional European countries, extending beyond their traditional range [26–33]. This geographic expansion is largely attributed to increasingly hot and dry summer conditions, a consequence of global climate change [3, 5].

Biologically, *Hyalomma* ticks are ditropic, meaning they utilize two hosts during their life cycle. After hatching, larvae typically remain on a single host to feed and develop into nymphs. Once engorged, the nymphs detach and seek a second host for their adult feeding stage [34, 35]. Larvae and nymphs commonly parasitize small mammals, hares, and birds, while adults preferentially feed on larger hoofed animals such as camels, cattle, deer, goats, horses, sheep, and wild boar. Humans may also serve as incidental hosts [34, 36].

To date, 15 *Hyalomma* species have been reported to carry CCHFV globally, including *Hyalomma aegyptium, H. anatolicum, H. asiaticum, H. dromedarii, H. excavatum, H. impeltatum, H. impressum, H. lusitanicum, H. marginatum, H. nitidum, H. rufipes, H. schulzei, H. scupense, H. truncatum*, and *H. turanicum*. Among these, eight species—*Hyalomma anatolicum*, *H. asiaticum*, *H. dromedarii*, *H. impeltatum*, *H. marginatum*, *H. rufipes*, *H. truncatum*, and *H. turanicum*—are considered to have a particularly high capacity for acquiring, maintaining, and transmitting the virus [7, 9, 37–40].

Beyond CCHFV, *Hyalomma* ticks are known to transmit a range of other zoonotic pathogens, including West Nile virus, Dhori virus, *Coxiella burnetii*, *Rickettsia aeschlimannii*, and *Borrelia turcica*, further emphasizing their significance in both public and veterinary health [9].

Among the species with high vector competence, *H. marginatum* is especially notable because of its ecological adaptability. Unlike most *Hyalomma* species, it is considered a generalist, capable of establishing populations without strict host dependence, although it does exhibit regional host preferences [41, 42]. Its abundance is more closely tied to the density of preferred local hosts than to its developmental stage. Moreover, immature *H. marginatum* ticks can disperse over long distances via migratory birds, particularly during spring migrations [27, 43, 44].

The rapidly changing climate is enabling *Hyalomma* ticks to complete their life cycle and reach reproductive maturity in increasingly broader areas of Europe. Consequently, both *Hyalomma* ticks and CCHF have been detected in more EU/EEA countries, as reflected in recent data from the European Centre for Disease Prevention and Control (ECDC) [45, 46]. Nevertheless, these data may underestimate the true distribution of both because of underreporting and limited surveillance. Therefore, their expanding range and role as vectors of high-consequence pathogens underscore the urgent need to evaluate potential implications for human and animal health. Hence, a comprehensive understanding of the current distribution and epidemiology of *Hyalomma* ticks and CCHFV is essential for effective public health preparedness.

To support this need, the present study aimed to systematically compile data on the first records of *Hyalomma* ticks, CCHFV, and CCHF disease in European countries through a review of peer-reviewed literature. The findings will contribute to evidence-based decision making for surveillance, forecasting, and preparedness against future CCHF outbreaks in both animals and humans.

## Methods

### Study design

The systematic literature review was designed according to the Preferred Reporting Items for Systematic Reviews and Meta-Analyses Statements (PRISMA) as described in Moher et al. [47]. These PRISMA guidelines were used to obtain unbiased results (Fig. S1). Search strings were primarily designed to cover peer-reviewed published literature about *Hyalomma* ticks being reported for the first time, irrespective of geographical origin. Later, the scientific articles collected were refined according to their study location, type of tick species, type of disease, and their novelty in terms of reporting. Finally, articles that reported the *Hyalomma* ticks, CCHF, and/or CCHFV for the first time in a European country were included in the study. The geographical scope of this study included all sovereign nations fully within geographical Europe, as well as partially recognized Kosovo, five countries with partial territories in Europe (Russia, Kazakhstan, Georgia, Azerbaijan, and Turkey), and two Asian countries that participate in European political institutions (Cyprus and Armenia).

### Literature search

The literature search was conducted in close collaboration between the participating researchers and librarians at the Swedish University of Agricultural Sciences (SLU). Keywords and search strings were defined and used for a global search in six online international databases: the Web of Science Core Collection, BIOSIS, Medline, CAB Abstracts, Zoological Record, and Scopus. The search string for the databases in the Web of Science [i.e. the Web of Science Core Collection, BIOSIS, MEDLINE, CAB Abstracts, Zoological Record) was “TS = ((tick OR ticks) AND (first* OR initial* OR primary* OR earliest*) AND (finding* OR record* OR report* OR manifest* OR detect* OR identif* OR discover* OR spott* OR evidence* OR assessment*) AND (Hyalomma*)]”.

The corresponding search performed in Scopus was “TITLE-ABS-KEY ((tick OR ticks) AND (first* OR initial* OR primary* OR earliest*) AND (finding* OR record* OR report* OR manifest* OR detect* OR identif* OR discover* OR spott* OR evidence* OR assessment*) AND (Hyalomma*))”. No restrictions on year were used in the search parameters for any of the databases. However, the university licence at SLU covers different years for different databases (Table 1). Articles retrieved were saved in EndNote-20 on Author, Title, and Year. Later, the saved articles were further screened out based on different criteria deemed relevant for this review (e.g. geographical origin, publication year, tick species, and type of pathogen). Finally, the selected articles were analysed to extract and compile information according to the study aims.

**Table 1:** Databases searched and the number of peer-reviewed scientific articles collected in January 2024

Name of the database	Years covered	Number of references
Web of Science Core Collection	1945–2023	462
Biosis	2009–2023	331
Medline	1950–2023	405
CAB Abstracts	1910–2023	654
Zoological Record	1990–2023	332
Scopus	1970–2023	536
Total		2720
Duplicates		1485
Unique references (after primary duplicate removal)		1235

### Literature screening

A total of 2720 references were identified and saved before duplicate sorting (Fig. S1). After duplicate sorting, 1485 articles were removed, and the remaining 1235 unique references were further screened following five different steps:

#### Step 1

The articles with non-European study origins, those published before 1970 or duplicated (the authors' names or titles were written in different ways, e.g. with initials or printed first names, using synonyms in the title, written in bold letters with little alteration of word order, etc.) were excluded based on title screening. The number of articles excluded after step 1 was 727, with the remaining 508 as unique references.

#### Step 2

At this step, articles without ‘*Hyalomma*’, ‘Crimean-Congo haemorrhagic fever’, ‘Crimean haemorrhagic fever’, ‘Congo fever’, ‘CCHF’, ‘CHF’, ‘CF’, ‘Crimean-Congo haemorrhagic fever virus’, ‘Crimean haemorrhagic fever virus’, ‘Congo fever virus’, ‘CCHFV’, ‘CHFV’, or ‘CFV’ in the title were excluded. After this second step, 256 articles were identified as unique references, while 252 were excluded.

#### Step 3

The remaining 256 articles were imported to ‘Rayyan’, an intelligent, systematic review management platform (https://www.rayyan.ai/), and rechecked for duplicates, with 12 more references being excluded. After removing the duplicates, the abstracts of the remaining 244 were evaluated blindly by the four authors for inclusion or exclusion based on the criteria listed in Table 2.

**Table 2:** Evaluation of abstracts (*n* = 244) by using inclusion and exclusion criteria relevant to the study

Inclusion criteria	Exclusion criteria
Studied *Hyalomma* spp.	Studied other species
Studied Crimean-Congo haemorrhagic fever virus	Studied other pathogens
Studied Crimean-Congo haemorrhagic fever disease	Studied other disease
Study origin was in Europe	Non-European study
First time the *Hyalomma* spp. and/or Crimean-Congo haemorrhagic fever and/or Crimean-Congo haemorrhagic fever virus was reported	Repeated reporting of the *Hyalomma* spp. and/or Crimean-Congo haemorrhagic fever and/or Crimean-Congo haemorrhagic fever virus

#### Step 4

Following blind reviews of abstracts and consensus among the four authors, 178 articles were excluded, and 66 articles were considered for full-text screening. However, of the 66 articles, 14 were written in languages other than English: Russian (*n* = 4); Turkish (*n* = 4); Spanish (*n* = 3); Polish (*n* = 1); Serbian (*n* = 1); Croatian (*n* = 1). These were, therefore, excluded, with a total of 52 articles used for full-text screening and a total of 192 excluded.

#### Step 5

Full text screening focused on whether the article provided information on one or more of the following topics: (1) First reporting year of *Hyalomma* spp.; (2) first reporting year of Crimean-Congo haemorrhagic fever virus (CHFV or CCHFV); (3) first human case of Crimean-Congo haemorrhagic fever (CHF or CCHF) disease.

The screening identified a total of 12 articles directly or indirectly matching the above-mentioned criteria (Fig. S1, Dataset S1), and the remaining 40 articles did not provide clear information about the first reporting of *Hyalomma* spp., CCHFV, or CCHF.

Of the 12 articles, 8 reported novel cases of either the *Hyalomma* ticks or CCHFV and/or CCHF in Armenia [48], the Czech Republic, Finland, Portugal [49], Germany [50], Hungary [8], Luxembourg [51], Spain [52], Sweden [5], and Turkey [53]. The remaining four articles included some relevant references but did not provide sufficient information to cover the other countries.

### Literature search beyond a systematic approach

Given the limited literature available on the predefined study objectives through the initial systematic search, an extended literature search was conducted in Google Scholar. References cited within the articles initially selected were screened for relevant references, and the reference lists of these new articles were also reviewed for additional information. Additionally, general review articles and their references to *Hyalomma* ticks and/or CCHFV and/or CCHF were considered. Articles not accessible via databases or internet search engines written in non-English languages were not directly cited. Instead, relevant information from these sources was referenced. This deep search following citation threads within the literature and targeted searching in Google Scholar resulted in 80 unique references. Further information was obtained using data collected and provided by the European network for medical and veterinary entomology (VectorNet). VectorNet, which started in 2014, has collected data from multiple sources on the presence, introduction, and absence of arthropod vector species of major concern to human and animal health [54]. VectorNet data were used to assess the status of countries for which information had not been found in the literature search.

## Results

### First finding of *Hyalomma* ticks

**Fig. 1:**
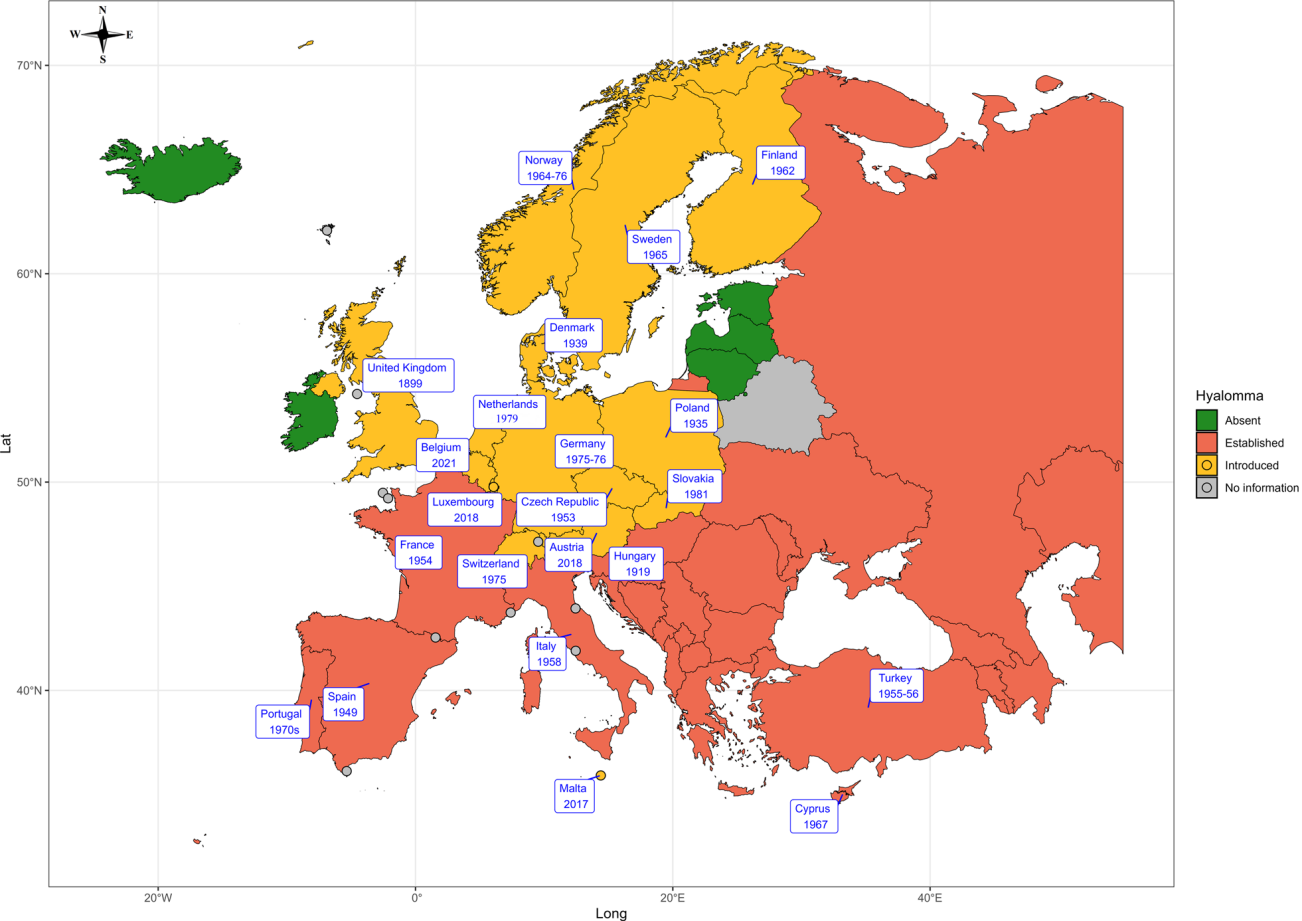
Geographic illustration depicting the earliest recorded presence of *Hyalomma* ticks in various European countries

*Hyalomma* ticks (one or several species of *Hyalomma*) are either endemic or have been detected at least once in 40 European countries. Table 3 (and Fig. 1) shows the first reporting year of *Hyalomma* ticks in different European countries. *Hyalomma* ticks are endemic in Albania, Armenia, Azerbaijan, Bosnia and Herzegovina, Bulgaria, Croatia, Georgia, Greece, Kazakhstan, Kosovo, Moldova, Montenegro, North Macedonia, Romania, Russia, Serbia, Slovenia, and Ukraine, but no specific information on the year of first detection is available for these countries. Also, there are no reported cases or no information available for Andorra, Belarus, Estonia, Iceland, Ireland, Latvia, Liechtenstein, Lithuania, Monaco, San Marino, and Vatican City.

**Table 3:** First year of *Hyalomma* ticks being reported in European countries

Country	First reporting year of *Hyalomma* spp.	Life stage of *Hyalomma* spp.	References
Austria	2018	Adult (male)	[55]
Belgium	2021		[56]
Cyprus	1967	Nymphs and larvae	[57]
Czech Republic	1953	Nymphs	[49]
Denmark	1939	Adult (male)	[26]
Finland	1962	Nymphs	[58]
France	1954	Unknown	[33]
Germany	1975–76	Unknown	[59]
Hungary	1919	Unknown	[60]
Italy	1958	Adult	[61]
Luxembourg	2018	Adult (male)	[51]
Malta	2017	Unknown	[62]
The Netherlands	1979	Unknown	[63]
Norway	1964–76	Unknown	[64]
Poland	1935	Adult (male)	[65]
Portugal	1970s	Unknown	[66]
Slovakia	1981	Adult (female)	[67]
Spain	1949	Nymphs and larvae	[69]
Sweden	1920s	Adults and nymphs	[26]
Switzerland	1975	Larvae	[71]
Turkey	1955–56	Unknown	[72]
UK	1899	Adult (female)	[73]
Albania, Armenia, Azerbaijan, Bosnia and Herzegovina, Bulgaria, Croatia, Georgia, Greece, Kazakhstan, Kosovo, Moldova, Montenegro, North Macedonia, Romania, Russia, Serbia, Slovenia, and Ukraine	Endemic, but no specific information available about the first year of detection		
Estonia, Iceland, Ireland, Latvia, and Lithuania	Absent		[56]
Andorra, Belarus, Liechtenstein, Monaco, San Marino, and Vatican City	Unknown/no information available

The first reporting year of *Hyalomma* ticks in the remaining countries is as follows:

Austria: In 2018, a male *H. marginatum* was detected for the first time on a horse in Lower Austria with no travel history, suggesting introduction via migratory birds. The tick tested negative for CCHFV but carried *Rickettsia aeschlimannii* [55].

Belgium: A 2021 VectorNet survey reported *H. marginatum* in Belgium, although details on life stage, gender, or host were not available [56].

Cyprus: In 1967, *H. marginatum* larvae and nymphs were found on 115 migratory birds representing 29 species, confirming its presence on the island [57].

The Czech Republic: The first record was in 1953 with four nymphs of *H. marginatum* from migratory birds. In 2019, a male *H. rufipes* was found on a native horse in South Moravia [49].

Denmark: The first report of an adult *H. marginatum* (male) came from the Danish island of Bornholm in early June 1939, likely transported as a nymph on a migratory bird from the Mediterranean or Africa [26].

Finland: *Hyalomma marginatum* (nymphs) was first recorded in the spring and summer of 1962 on migratory birds returning from Africa, southern Europe, and Asia in two island groups in Finland [58].

France: *Hyalomma* ticks have long been established in southern France, with the first report dating back to 1954 in the Camargue (*H. suspense*). Historical data also indicate the presence of *H. marginatum* in southern France between the 1940 s and 1980 s [33].

Germany: The first report was in 1975–1976 from migratory birds on Helgoland Island [59]. In 2006, a female *H. marginatum* was found on a person in southern Germany, although its origin—local or imported—remains unclear [50].

Hungary: The first mention of *Hyalomma* ticks in Hungary dates to 1919 by Kolan [60]. Identification of the specific *Hyalomma* species and tick life stage was hindered by limited access to the full article.

Italy: *Hyalomma* ticks have been endemic to Italy since ancient times, particularly in Sardinia, Sicily, and several smaller islands along the Italian coast. However, the earliest documented evidence of their presence dates back to the 1950 s [61]. The specific species of *Hyalomma* was not identified because of limited access to the full article.

Luxembourg: On 31 August 2018, a male *H. marginatum* was collected in the city of Dudelange, marking the first recorded case of this species in the country [51]. Additional specimens were found on horses in 2020, with genetic analysis suggesting passive introduction via migratory birds [51].

Malta: *Hyalomma marginatum* was first identified as a new *Ixodidae* species in Malta by Pfliegler et al. in 2017 [62], with specimens collected from rabbits. However, an unpublished VectorNet survey in August 2016 identified *H. lusitanicum* and *H. marginatum* in Malta, although no details on their life stage or host were provided.

The Netherlands: The first documented *Hyalomma* species in The Netherlands was *H. aegyptium*, reported by Bronswijk et al. in 1979 on imported ticks [63]. Later, a male *H. marginatum* was identified during a tick survey of companion animals conducted between July 2005 and October 2006, following an outbreak of autochthonous canine babesiosis.

Norway: Between 1964–1976, 602 ticks were collected from birds on southern islands, with 3% identified as *H. marginatum*, likely transported from southern Europe or Africa [64].

Poland: An examination of tick specimens from the Natural History Department’s collection between 1930 and 1948 identified the first recorded presence of *H. marginatum* (a male) in Poland [65].

Portugal: The first report of *Hyalomma* ticks is from the 1970 s, collected from cattle in southern Portugal [66]. Due to limited access to the full article, the specific life stage of the tick was not available.

Slovakia: The first adult *H. marginatum* (a female) was reported in August 1981. It is thought to have developed from an engorged nymph transported by a migratory bird and was collected from vegetation at Topolovec, near Gabcikovo in the Danube region of southern Slovakia (then Czechoslovakia) [67].

Spain: Although Hoogstraal et al. [68] reported the presence of *H. marginatum* larvae and nymphs on Portuguese and Spanish migratory warblers caught in Egypt, the first mention of *Hyalomma* ticks in Spain was by Parker et al. [69], who collected 16 adult *H. savignyi* from a sheep in Bobeda, Salamanca, on 25 April 1949.

Sweden: Nymphs and males of *H. aegyptium* were frequently recorded on imported turtles in Sweden as early as the 1920 s [26]. *Hyalomma marginatum* was first reported on migratory birds at Öland’s Ottenby Bird Station in 1965 [70], with the first autochthonous adult cases of *H. marginatum* and *H. rufipes* documented in 2018–2019 on horses, cattle, and humans across 14 provinces [5].

Switzerland: The first report of *Hyalomma* ticks in Switzerland, published by Aeschlimann and Buttiker [71], mentioned that larvae of *H. marginatum* were brought into Switzerland by migratory birds on several occasions during the 1970 s or earlier.

Turkey: *Hyalomma* ticks are endemic in Turkey, with the first report available dating back to 1955–1956 when 996 cattle in Ankara and nearby villages were examined for blood parasites causing Theileria infection [72]. The specific species of *Hyalomma* and the life stage of the ticks were not accessible because of limited access to the full article.

United Kingdom: The earliest record was a female *Hyalomma syriacum* found in Surrey in 1899 [73]. More recently, *H. marginatum* larvae and nymphs were reported on migratory birds, and in 2018, a male *H. rufipes* was found on horses with no travel history [74].

### First reporting of CCHF and CCHFV

**Fig. 2:**
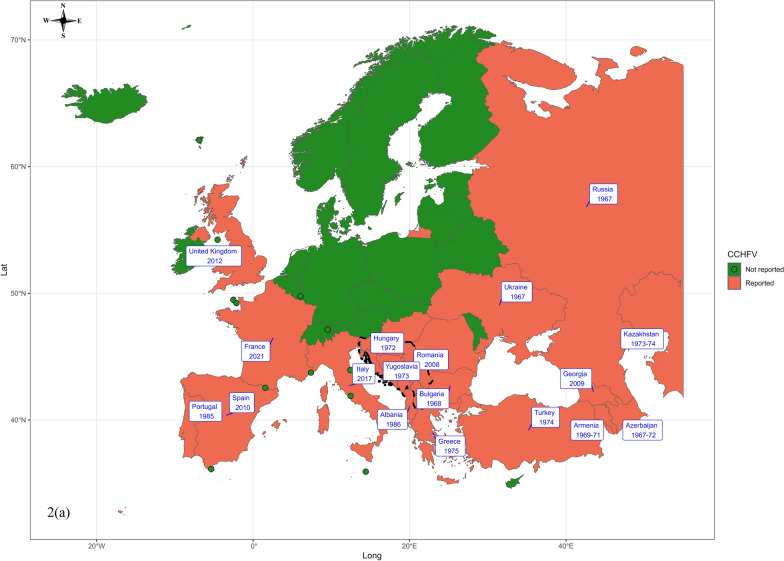

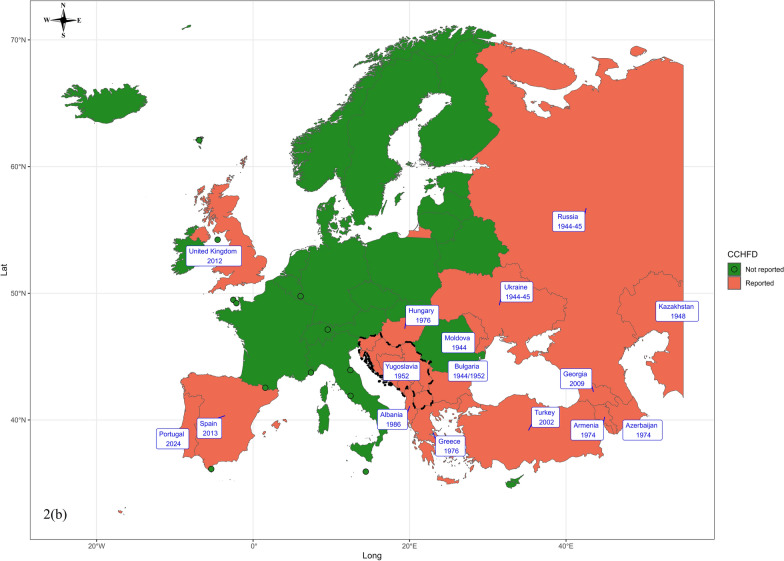
Geographic illustration depicting the earliest recorded presence of (**a**) Crimean-Congo haemorrhagic fever virus (CCHFV) and (**b**) Crimean-Congo haemorrhagic fever disease (CCHFD) in various European countries

CCHFV has been detected in 24 European countries and CCHF in 22 (Table 4, Fig. 2). A short summary of the first reporting of CCHFV and CCHF disease in these countries is as follows:

**Table 4:** First detection of Crimean-Congo haemorrhagic fever (CCHF) and Crimean-Congo haemorrhagic fever virus (CCHFV) in European countries

Country	First reporting year of CHFV/CCHFV	First human case of CHF/CCHF disease	Vector-disease detection time interval [in year(s)]	References
Albania	1986	1986	15	[75]
Armenia	1969–71	1974	2–5	[76, 77]
Azerbaijan	1967–1972	1974	2–7	[77, 78]
Bulgaria	1968	1952/1953	15	[77, 79]
France	2021–2022	–	NA	[80]
Georgia	2009	2009	0	[81]
Greece	1975	1976	1	[82–84]
Hungary	1972	1976/2004	4	[8, 85]
Italy	2017	–	NA	[86, 87]
Kazakhstan	1973–74	1948	25–26	[12]
Moldova	–	1944	NA	[88]
Portugal	1985	2024	39	[89, 90]
Romania	2008	–	NA	[91]
Russia	1967	1944–1945	22–23	[77]
Spain	2010	2013	3	[25, 52]
Turkey	1974	2002	28	[92, 93]
Ukraine	1967	1944–1945	22–23	[77]
UK	2012	2012	0	[94]
Bosnia and Herzegovina, Croatia, Macedonia, Montenegro, Serbia, Slovenia, and Kosovo	1973	1952	21	[95, 96]

Albania: The first confirmed report of CCHFV and CCHF disease in Albania, as published by Eltari et al. [75], was in lower-lying, hilly areas where livestock rearing and *H. marginatum* ticks on cattle were prevalent.

Armenia: The first confirmed report of CCHFV in Armenia was documented between 1969 and 1971 through antibody analysis. In a subsequent investigation in 1972, several strains of the virus were isolated from *H. marginatum* and *H. anatolicum* ticks collected on cattle in Soviet Armenia [76]. The first human case of CCHF in Armenia was serologically confirmed in 1974, although similar cases had been reported earlier [77].

Azerbaijan: Between 1967 and 1972, a seroepidemiological survey confirmed widespread CCHFV circulation. In 1972, ten virus strains were isolated from *H. marginatum* ticks—the first such record in the country. The first human outbreak occurred in 1974, involving 60 suspected cases [77, 78].

Bulgaria: Earlier data published in 1960 in Bulgarian by Ivanov indicate that the first CCHF case in Bulgaria was identified in 1944 in the Razgrad and Kolarovograd areas, and the CCHFV vector was tick-infested horses or contaminated fodder from the Soviet army from World War II [77]. However, Papa et al. [79] state that it was first recognized in the country in 1952 and became a reportable disease in 1953. The first CCHFV isolation in Bulgaria was reported by Vasilenko and his team in 1968 from the blood samples of a human patient [77].

France: In France, antibodies against CCHFV were identified for the first time in 2021–2022 in cattle, and the virus was isolated in 2022 in ticks of the species *H. marginatum* collected from cattle in the Occitanie region [80]. No human case of CCHF has been reported so far.

Georgia: Serological analysis on 9 September 2009 confirmed the presence of CCHF antibodies in Georgia, marking the first report of CCHFV in the country. The serum was collected from a 30 year-old male patient from a suburb of Tbilisi, Georgia, who was admitted to the hospital on 25 August 2009 and later diagnosed with CCHF, the first CCHF case in Georgia [81].

Greece: CCHFV was first isolated in Greece in May 1975 from *Rhipicephalus bursa* ticks collected from goats in Vergina, Macedonia [82]. In 1976, a veterinarian acquired an asymptomatic laboratory infection while working with the virus—the first reported human case [83]. Natural human infections were later confirmed through serological evidence in northern Greece in 1980–1981 [84].

Hungary: CCHFV was first isolated in Hungary in 1972, with seropositivity detected in cattle and sheep the following year. The first confirmed human case occurred in 2004 [8]. However, earlier serological evidence by Horváth [85] showed antibodies in 17 of 587 individuals working with animals, including one in a Budapest slaughterhouse, suggesting possible earlier human exposure.

Italy: In April 2017, Mancuso et al. [86] detected CCHFV in an *H. rufipes* nymph from a migratory bird (*Saxicola rubetra*, whinchat) on Ventotene Island, marking the first evidence of the virus in Italy. Additionally, anti-CCHFV antibodies in cattle sera from the Basilicata region in 2021 indicate the first local CCHFV presence and bovine infections [87]. No human case of CCHF has been reported to date in Italy.

Kazakhstan: In Kazakhstan, locals knew of CCHF as “Coc-ala” for many decades because of its haemorrhagic skin manifestations, with the first official reports in 1948 documenting an outbreak affecting six farmers, half of whom died. A serosurveillance programme in 1973–1974 found evidence of CCHFV in humans and animals across South Kazakhstan and Kyzylorda. This provided the first evidence of CCHFV circulation in Kazakhstan [12].

Moldova: CCHF disease was first detected in 1944 in the former Union of Soviet Socialist Republics (USSR), including Moldova [88]. However, limited information is available about the specific cases and whether the virus was later isolated.

Portugal: In the 1980 s, a serological survey detected CCHFV antibodies in two human sera, indicating virus circulation in southern Portugal [89]. The first confirmed human case occurred on 14 August 2024 in an 80 year-old man from Bragança with no travel history. He developed symptoms on 11 July and later died. No additional cases or symptomatic contacts were reported [90].

Romania: A total of 471 serum samples from sheep in Tulcea, Northern Dobrogea, were collected between June and September 2008 and tested for antibodies specific to CCHFV. Of these, 131 samples (27.8%) tested positive, providing the first evidence of CCHFV circulation in Romania [91]. No human case of CCHF has been reported to date.

Russia: CCHF is prevalent in Russian regions bordering the Caspian Sea, Black Sea, and Sea of Azov. The first recorded case of CCHF in the modern Russian Federation was in the Krasnodar region, with a few cases observed on the Taman Peninsula during the 1944 epidemic [77]. The virus was first isolated from the sera of a CCHF patient by Chumakov and his colleagues in 1967 at the Institute of Poliomyelitis and Viral Encephalitides, Moscow [77].

Spain: CCHFV was first reported in Spain in November 2010, identified via PCR in *H. lusitanicum* ticks from red deer in Cáceres. The strains showed 98% similarity to those from Mauritania and Senegal, suggesting introduction via migratory birds [25]. Although many human cases have been reported in Spain since 2013 [45], a retrospectively confirmed case from 2013 is now considered the earliest known human infection in the country [52].

Turkey: A 1974 seroepidemiological study in Turkey detected anti-CCHFV antibodies in 26 of 1100 human sera, indicating virus presence despite no clinical cases at the time [92]. The first confirmed cases occurred in 2002–2003, with 19 suspected cases, six positives, and two virus isolates genetically related to strains from the former Yugoslavia and southwestern Russia. The fatality rate was 20%, and haemophagocytosis was observed in half of the patients [93]. In 2005, CCHFV was first detected in ticks through genetic analysis [53].

Ukraine: The first identification of CCHFV in Ukraine was initially presumed to be around 1967 after its first isolation in Moscow, but Hoogstraal [77] reported that it was in 1972–73 when 33 strains were isolated from ticks in various localities across Crimea. Although an earlier report from 1854 mentioned a similar disease called ‘malignant Crimean fever’ in the Ukrainian SSR, CCHF was first officially recognized between June and September 1944 in the Crimean Peninsula when 92 military personnel were hospitalized and nine of them died [77].

United Kingdom: In October 2012, a 38-year-old Afghan male returned to Glasgow from Afghanistan and developed symptoms of CCHF disease. Blood and urine samples confirmed CCHFV infection via PCR testing within 36 h. The likely source was exposure to a slaughtered calf at a wedding in Afghanistan. Despite treatment, the patient deteriorated and died 96 h after admission. This was the first confirmed case of CCHF and CCHFV in the UK [94].

Former members of the Socialist Federal Republic of Yugoslavia (SFRY): The first isolation of three strains of CCHFV in 1973 from ticks in Tetovo, Macedonia, retrospectively confirmed the 1970 outbreak in the area as CCHF disease. This finding provides evidence of the presence of CCHFV in the former SFRY (i.e. Bosnia and Herzegovina, Croatia, Macedonia, Montenegro, Serbia, Slovenia, and Kosovo) since 1973 [95, 96]. Additionally, Vesenjak-Hiran et al. [96] reported outbreaks of CCHF disease in the former SFRY dating back to 1952.

In addition, the virus is presumed to be present in Moldova because of a historical prevalence of human CCHF cases [88], although no accessible information is available. Furthermore, no information is available for Andorra, Belarus, Liechtenstein, Monaco, San Marino, and Vatican City, as no CCHFV has been isolated or no accessible literature has reported CCHF cases in other European countries.

## Discussion

The distribution of *Hyalomma* ticks across Europe has undergone significant changes over the past century, influenced by ecological, climatic, and anthropogenic factors [5, 35]. A chronological overview reveals distinct phases of spread:

The earliest documented occurrences of *Hyalomma* ticks in Europe date back to the late nineteenth and early twentieth centuries (Table 3), with the UK reporting *Hyalomma* ticks in 1899, followed by Hungary in 1919 [60, 73]. Other countries, including Sweden, Poland, and Denmark, recorded cases between the 1920 s and 1940 s, often linked to imported animals and migratory birds [26, 65]. These early findings reflect both the limited surveillance capacity of the time and the anecdotal nature of observations in non-endemic areas. Notably, endemic countries such as Italy lacked formal records during this period.

Reports increased across Europe, with Spain, Italy, and the Czech Republic documenting tick presence in the 1950 s, followed by France and Turkey [33, 49, 61, 69, 72]. During this period, the role of migratory birds in transporting immature ticks (larvae and nymphs) became more evident, particularly in northern regions such as Sweden, Finland, Norway, and Cyprus [57, 58, 64, 70].

During the twentieth century (1970–2000), the spread continued, with new reports from Switzerland, Portugal, Germany, Slovakia, and The Netherlands [59, 63, 66, 67, 71], indicating ongoing expansion across the continent.

In the twenty-first century, further documentation emerged from Malta, Austria, Luxembourg, and Belgium [51, 55, 56, 62]. These cases often involved migratory birds and imported animals, underscoring the dynamic nature of tick distribution.

The distribution of *Hyalomma* ticks across Europe is regionally variable and closely linked to climatic conditions and ecological pathways. Southern European countries such as Italy, Spain, and France host long-established tick populations, largely because of their warmer climates and proximity to major migratory bird routes [27, 43]. In contrast, Central and Eastern European countries—including Hungary, the Czech Republic, and Slovakia—have primarily reported immature ticks (larvae and nymphs), often associated with bird migration [49, 60, 67].

In Northern and Western Europe, including Sweden, Finland, Norway, the UK, Belgium, and The Netherlands, *Hyalomma* ticks are typically introduced via migratory birds and, to a lesser extent, through imported animals. While most detections involve immature stages, recent climate shifts have facilitated local establishment in some areas [5].

Our study disentangles a two-phase model of tick establishment: first, the introduction of larvae and nymphs via migratory birds; second, the development of adult populations enabled by suitable climatic conditions and the presence of appropriate host animals. Larvae are predominantly transported by birds, whereas nymphs require specific hosts and environmental conditions to moult into adults. Nymphs feed on small vertebrates such as rodents and birds during spring and summer, while adult ticks feed and reproduce on larger mammals, including cattle, goats, horses, sheep, deer, wild boar, and occasionally humans [34].

The emergence and spread of CCHF and CCHFV in Europe represent a growing public health concern, despite currently low prevalence in most regions. Surveillance remains essential to monitor viral circulation and assess future risks.

Historical records show that CCHFV and CCHF have been present in southeastern Europe since the mid-twentieth century [77]. Initial reports from the 1940 s include Ukraine, Moldova, Russia, and Kazakhstan [12, 77, 88]. Bulgaria and the former SFR of Yugoslavia followed in the 1950 s [77, 96], with additional detections in Armenia, Azerbaijan, Greece, Hungary, and Turkey during the 1960 s and 1970 s [76–78, 82–85, 92].

The 1980 s brought new cases from Albania and Portugal, while the 1990 s saw no significant additions [75, 89]. In the twenty-first century, Georgia reported cases in the 2000 s, followed by Italy, Spain, and the UK in the 2010 s [25, 81, 86, 94]. Most recently, France documented its first CCHFV cases, and Portugal reported its first confirmed CCHF cases in the 2020 s [80, 90]. These findings indicate that while southeastern Europe remains the primary endemic region, southwestern Europe—including France, Italy, Portugal, and Spain—is increasingly affected.

Although no confirmed cases have been reported in northern Europe, the recent detection of CCHFV antibodies in ruminant sera from parts of southern Europe [80] suggests active viral circulation and the presence of *Hyalomma* ticks. These findings underscore the importance of continued entomological and epidemiological surveillance, even in regions where the virus has not yet been isolated or human cases have not occurred.

Variations in the detection and reporting of CCHFV and CCHF across Europe are shaped by differences in surveillance systems, ecological conditions, and livestock practices [11, 45]. Migratory birds and imported animals (e.g. livestock, pets) contribute to the spread of both ticks and the virus [25]. Climate change further exacerbates these dynamics by expanding tick habitats, increasing tick activity and reproduction, and altering migratory patterns [5]. Human travel also plays a role, with eight imported CCHF cases reported in Europe between 1980 and 2024, including one in the UK following travel to Afghanistan [94, 97].

Historically, tick surveillance relied on tick dragging—a localized method involving cloth dragged over vegetation to collect host-seeking ticks. While effective for assessing local abundance and endemic species, this technique is limited in detecting newly introduced or low-density populations [98, 99]. In recent years, citizen science and digital tools (e.g. mobile apps, online platforms) have expanded the spatial and temporal scope of surveillance, enhancing early warning systems—as seen in Czechia, Hungary, and Sweden, where citizen reports led to the detection of emerging *Hyalomma* ticks [5, 98, 99].

While initial detections often mark the first formal documentation of the ticks, virus, or disease, they rarely represent the true onset of their presence. In many cases, introduction and establishment may precede reporting by months or even years because of surveillance gaps, inconsistent national reporting systems, and limited field investigations. This underreporting contributes to an incomplete understanding of the actual distribution and prevalence of both the vector and the virus.

Given ongoing environmental changes and shifting vector distributions, a One Health approach—integrating human, animal, and environmental health—is essential. Countries without established *Hyalomma* populations or CCHF cases can benefit from the experiences of neighbouring nations by adopting robust surveillance systems and crisis response plans. Cross-border collaboration and simulation exercises can further strengthen preparedness, allowing authorities to test interventions and harmonize responses before an outbreak occurs.

## Conclusions

This study highlights the evolving epidemiology of CCHF in Europe and contributes to improved risk mapping of *Hyalomma* ticks and CCHFV. It underscores the need for expanded surveillance in emerging risk areas and supports integrated One Health approaches linking entomology, virology, and human and animal health. The findings may inform public and animal health strategies, particularly in the context of climate-driven changes in vector ecology, where coordinated and forward-looking efforts are essential to mitigate future risks.

## Supplementary Information

Supplementary Material 1: Fig. S1. Preferred Reporting Items for Systematic Reviews and Meta-Analysesflow diagram illustrating the stages of the literature search process

Supplementary Material 2: Dataset S1. Articles retrieved through a systematic literature search that provided relevant information and references on *Hyalomma*, CCHF, or CCHFV for the target countries.

Supplementary Material 3: PRISMA checklist

## Data Availability

All data analysed in this systematic review were obtained through a comprehensive literature search and are presented in the tables and figures within the manuscript. No additional data or materials are available for sharing.

## Abbreviations

CCHF: Crimean-Congo haemorrhagic fever
CCHFV: Crimean-Congo haemorrhagic fever virus
ECDC: European Centre for Disease Prevention and Control
SFRY: Socialist Federal Republic of Yugoslavia
USSR: Union of Soviet Socialist Republics

## References

[1] Maqbool M, Sajid MS, Saqib M, Anjum FR, Tayyab MH, Rizwan HM, et al. Potential mechanisms of transmission of tick-borne viruses at the virus-tick interface. Front Microbiol. 2022;13:846884. 10.3389/fmicb.2022.846884. 35602013 10.3389/fmicb.2022.846884 PMC9121816

[2] Semenza JC, Paz S. Climate change and infectious disease in Europe: impact, projection and adaptation. Lancet Reg Health. 2021;9:100230. 10.1016/j.lanepe.2021.100230. 10.1016/j.lanepe.2021.100230 PMC8513157 34664039

[3] Chitimia-Dobler L, Schaper S, Rieß R, Bitterwolf K, Frangoulidis D, Bestehorn M, et al. Imported *Hyalomma* ticks in Germany in 2018. Parasit Vectors. 2019;12:134. 10.1186/s13071-019-3380-4. 30909964 10.1186/s13071-019-3380-4 PMC6434826

[4] Estrada-Peña A, Ayllón N, De La Fuente J. Impact of climate trends on tick-borne pathogen transmission. Front Physiol. 2012;3:64. 10.3389/fphys.2012.00064. 22470348 10.3389/fphys.2012.00064 PMC3313475

[5] Grandi G, Chitimia-Dobler L, Choklikitumnuey P, Strube C, Springer A, Albihn A, et al. First records of adult *Hyalomma marginatum* and *H. rufipes* ticks (Acari: Ixodidae) in Sweden. Ticks Tick Borne Dis. 2020;11:101403. 10.1016/j.ttbdis.2020.101403. 32037097 10.1016/j.ttbdis.2020.101403

[6] Negredo A, de la Calle-Prieto F, Palencia-Herrejón E, et al. Autochthonous Crimean-Congo hemorrhagic fever in Spain. N Engl J Med. 2017;377:154–61. 10.1056/NEJMoa1615162. 28700843 10.1056/NEJMoa1615162

[7] Gargili A, Estrada-Peña A, Spengler JR, Lukashev A, Nuttall PA, Bente DA. The role of ticks in the maintenance and transmission of Crimean-Congo hemorrhagic fever virus: a review of published field and laboratory studies. Antivir Res. 2017;144:93–119. 10.1016/j.antiviral.2017.05.010. 28579441 10.1016/j.antiviral.2017.05.010 PMC6047067

[8] Hornok S, Horváth G. First report of adult *Hyalomma marginatum rufipes* (vector of Crimean-Congo haemorrhagic fever virus) on cattle under a continental climate in Hungary. Parasit Vectors. 2012;5:170. 10.1186/1756-3305-5-170. 22889105 10.1186/1756-3305-5-170 PMC3436687

[9] Kumar B, Manjunathachar HV, Ghosh S. A review on *Hyalomma* species infestations on human and animals and progress on management strategies. Heliyon. 2020;6:e05675. 10.1016/j.heliyon.2020.e05675. 33319114 10.1016/j.heliyon.2020.e05675 PMC7726666

[10] Robinson PM. *Theileriosis annulata* and its transmission—a review. Trop Anim Health Prod. 1982;14:3–12. 10.1007/BF02281092. 6805112 10.1007/BF02281092

[11] Bente DA, Forrester NL, Watts DM, McAuley AJ, Whitehouse CA, Bray M. Crimean-Congo hemorrhagic fever: history, epidemiology, pathogenesis, clinical syndrome and genetic diversity. Antivir Res. 2013;100:159–89. 10.1016/j.antiviral.2013.07.006. 23906741 10.1016/j.antiviral.2013.07.006

[12] Nurmakhanov T, Sansyzbaev Y, Atshabar B, Deryabin P, Kazakov S, Zholshorinov A, et al. Crimean-congo haemorrhagic fever virus in Kazakhstan (1948–2013). Int J Infect Dis. 2015;38:19–23. 10.1016/j.ijid.2015.07.007. 26183415 10.1016/j.ijid.2015.07.007

[13] World Health Organization (WHO). Crimean-Congo haemorrhagic fever. 2022. https://www.who.int/news-room/fact-sheets/detail/crimean-congo-haemorrhagic-fever.

[14] Al-Abri SS, Abaidani IA, Fazlalipour M. Current status of Crimean-Congo haemorrhagic fever in the World Health Organization Eastern Mediterranean Region: issues, challenges, and future directions. Int J Infect Dis. 2017;58:82–9. 10.1016/j.ijid.2017.02.018. 28259724 10.1016/j.ijid.2017.02.018 PMC7110796

[15] Ergonul O. Crimean-Congo haemorrhagic fever. Lancet Infect Dis. 2006;6:203–14. 10.1016/S1473-3099(06)70435-2. 16554245 10.1016/S1473-3099(06)70435-2 PMC7185836

[16] Sidira P, Maltezou HC, Haidich AB, Papa A. Seroepidemiological study of Crimean-Congo haemorrhagic fever in Greece, 2009–2010. Clin Microbiol Infect. 2012;18:E16-9. 10.1111/j.1469-0691.2011.03718.x. 22192082 10.1111/j.1469-0691.2011.03718.x

[17] Yadav PD, Patil DY, Shete AM, Kokate P, Goyal P, Jadhav S. Nosocomial infection of CCHF among health care workers in Rajasthan, India. BMC Infect Dis. 2016;16:624. 10.1186/s12879-016-1971-7. 27809807 10.1186/s12879-016-1971-7 PMC5094004

[18] European Food Safety Authority (EFSA). EFSA panel on animal health and welfare: scientific opinion on the role of tick vectors in the epidemiology of Crimean-Congo hemorrhagic fever and African swine fever in Eurasia. EFSA J. 2010;8:1703. 10.2903/j.efsa.2010.1703.

[19] Ahmeti S, Berisha L, Halili B, Ahmeti F, von Possel R, Thomé-Bolduan C, et al. Crimean-Congo hemorrhagic fever, Kosovo, 2013–2016. Emerg Infect Dis. 2019;25:321–4. 10.3201/eid2502.171999. 30666932 10.3201/eid2502.171999 PMC6346452

[20] Ergonul O, Celikbas A, Yildirim U, Zenciroglu A, Erdogan D, Ziraman I, et al. Pregnancy and Crimean-congo haemorrhagic fever. Clin Microbiol Infect. 2010;16:647–50. 19778302 10.1111/j.1469-0691.2009.02905.x

[21] Matser A, Hartemink N, Heesterbeek H, Galvani A, Davis S. Elasticity analysis in epidemiology: an application to tick-borne infections. Ecol Lett. 2009;12:1298–305. 10.1111/j.1461-0248.2009.01378.x. 19740112 10.1111/j.1461-0248.2009.01378.x

[22] Gunes T, Engin A, Poyraz O, Elaldi N, Kaya S, Dokmetas I, et al. Crimean-congo hemorrhagic fever virus in high-risk population, Turkey. Emerg Infect Dis. 2009;15:461–4. 10.3201/eid1503.080687. 19239765 10.3201/eid1503.080687 PMC2681111

[23] Pshenichnaya NY, Nenadskaya SA. Probable Crimean-congo hemorrhagic fever virus transmission occurred after aerosol-generating medical procedures in Russia: nosocomial cluster. Int J Infect Dis. 2015;33:120–2. 10.1016/j.ijid.2014.12.047. 25576827 10.1016/j.ijid.2014.12.047

[24] Tsergouli K, Karampatakis T, Haidich AB, Metallidis S, Papa A. Nosocomial infections caused by Crimean-Congo haemorrhagic fever virus. J Hosp Infect. 2020;105:43–52. 10.1016/j.jhin.2019.12.001. 31821852 10.1016/j.jhin.2019.12.001

[25] Estrada-Peña A, Palomar AM, Santibáñez P, Sánchez N, Habela MA, Portillo A, et al. Crimean-congo hemorrhagic fever virus in ticks, Southwestern Europe, 2010. Emerg Infect Dis. 2012;18:179–80. 10.3201/eid1801.111040. 22261502 10.3201/eid1801.111040 PMC3310114

[26] Jaenson TGT, TäLleklint L, Lundqvist L, Olsen B, Chirico J, Mejlon H. Geographical distribution, host associations, and vector roles of ticks (Acari: Ixodidae, Argasidae) in Sweden. J Med Entomol. 1994;31:240–56. 10.1093/jmedent/31.2.240. 8189415 10.1093/jmedent/31.2.240 PMC7107449

[27] Jameson LJ, Morgan PJ, Medlock JM, Watola G, Vaux AG. Importation of *Hyalomma marginatum*, vector of Crimean-Congo haemorrhagic fever virus, into the United Kingdom by migratory birds. Ticks Tick-borne Dis. 2012;3:95–9. 10.1016/j.ttbdis.2011.12.002. 22300969 10.1016/j.ttbdis.2011.12.002

[28] Martyn KP. Provisional atlas of the ticks (Ixodea) of the British Isles. Huntingdon: Biological Records Centre, Institute of Terrestrial Ecology; 1988.

[29] Pascucci I, Di Domenico M, Capobianco Dondona G, Di Gennaro A, Polci A, Capobianco Dondona A, et al. Assessing the role of migratory birds in the introduction of ticks and tick-borne pathogens from African countries: an Italian experience. Ticks Tick Borne Dis. 2019;10:101272. 10.1016/j.ttbdis.2019.101272. 31481344 10.1016/j.ttbdis.2019.101272

[30] Rumer L, Graser E, Hillebrand T, Talaska T, Dautel H, Mediannikov O, et al. *Rickettsia aeschlimannii* in *Hyalomma marginatum* ticks, Germany. Emerg Infect Dis. 2011;17:325–6. 10.3201/eid1702.100308. 21291625 10.3201/eid1702.100308 PMC3204748

[31] Toma L, Mancini F, Di Luca M, Cecere JG, Bianchi R, Khoury C, et al. Detection of microbial agents in ticks collected from migratory birds in central Italy. Vector Borne Zoonotic Dis. 2014;14:199–205. 10.1089/vbz.2013.1458. 24576218 10.1089/vbz.2013.1458 PMC3952585

[32] Trilar T. Ticks (Acarina: Ixodidae) on birds in Slovenia. Acrocephalus. 2004;25:213–6.

[33] Vial L, Stachurski F, Leblond A, Huber K, Vourc’h G, René-Martellet M, et al. Strong evidence for the presence of the tick *Hyalomma marginatum* Koch, 1844 in southern continental France. Ticks Tick Borne Dis. 2016;7:1162–7. 10.1016/j.ttbdis.2016.08.002. 27568169 10.1016/j.ttbdis.2016.08.002

[34] Apanaskevich DA. Host-parasite relationships of the genus *Hyalomma* Koch, 1844 (Acari, Ixodidae) and their connection with microevolutionary process. Parazitologiia. 2004;38:515–23. 15656094

[35] Valcárcel F, González J, González MG, Sánchez M, Tercero JM, Elhachimi L, et al. Comparative ecology of *Hyalomma lusitanicum* and *Hyalomma marginatum* Koch, 1844 (Acarina: Ixodidae). Insects. 2020;11:303. 10.3390/insects11050303. 32414220 10.3390/insects11050303 PMC7290797

[36] Hoogstraal H. African Ixodea: ticks of the Sudan. 1956. 1: 388–503.

[37] Bakheit MA, Latif AA, Vatansever Z, Seitzer U, Ahmed J. The huge risks due to *Hyalomma* ticks. In: Mehlhorn H, editor. Arthropods as vectors of emerging diseases. Parasitology research monographs, vol. 3. Berlin: Springer; 2012. p. 149–56.

[38] Deka MA. Crimean-congo hemorrhagic fever geographic and environmental risk assessment in the Balkan and Anatolian Peninsulas. Pap Appl Geogr. 2018;4:46–71. 10.1080/23754931.2017.1378122.

[39] Mourya DT, Yadav PD, Shete AM, Gurav YK, Raut CG, Jadi RS, et al. Detection, isolation and confirmation of Crimean-Congo hemorrhagic fever virus in human, ticks and animals in Ahmadabad, India, 2010–2011. PLoS Negl Trop Dis. 2012;6:e1653. 10.1371/journal.pntd.0001653. 22616022 10.1371/journal.pntd.0001653 PMC3352827

[40] Spengler JR, Estrada-Peña A. Host preferences support the prominent role of *Hyalomma* ticks in the ecology of Crimean-Congo hemorrhagic fever. PLoS Negl Trop Dis. 2018;12:e0006248. 10.1371/journal.pntd.0006248. 29420542 10.1371/journal.pntd.0006248 PMC5821391

[41] Grech-Angelini S, Stachurski F, Lancelot R, et al. Ticks (Acari: Ixodidae) infesting cattle and some other domestic and wild hosts on the French Mediterranean island of Corsica. Parasit Vectors. 2016;9:582. 10.1186/s13071-016-1876-8. 27842608 10.1186/s13071-016-1876-8 PMC5109666

[42] Léger E, Vourc’h G, Vial L, Chevillon C, McCoy KD. Changing distributions of ticks: causes and consequences. Exp Appl Acarol. 2013;59:219–44. 10.1007/s10493-012-9615-0. 23015121 10.1007/s10493-012-9615-0

[43] Capek M, Literak I, Kocianova E, Sychra O, Najer T, Trnka A, et al. Ticks of the *Hyalomma marginatum* complex transported by migratory birds into Central Europe. Ticks Tick Borne Dis. 2014;5:489–93. 10.1016/j.ttbdis.2014.03.002. 24877976 10.1016/j.ttbdis.2014.03.002

[44] Hasle G, Bjune G, Edvardsen E, Jakobsen C, Linnehol B, Røer JE, et al. Transport of ticks by migratory passerine birds to Norway. J Parasitol. 2009;95:1342–51. 10.1645/GE-2146.1. 19658452 10.1645/GE-2146.1

[45] European Centre for Disease Prevention and Control (ECDC). Historical data on local transmission of Crimean-Congo haemorrhagic fever in the EU/EEA. Stockholm: ECDC; 2024. https://www.ecdc.europa.eu/en/infectious-disease-topics/crimean-congo-haemorrhagic-fever/surveillance-and-updates/local-transmission-eueea-previous-years.

[46] European Centre for Disease Prevention and Control (ECDC) and European Food Safety Authority (EFSA). Tick maps. Stockholm: ECDC. 2023. https://www.ecdc.europa.eu/en/disease-vectors/surveillance-and-disease-data/tick-maps.

[47] Moher D, Liberati A, Tetzlaff J, Altman DG, PRISMA Group. Preferred reporting items for systematic reviews and meta-analyses: The PRISMA statement. PLoS Med. 2009;6:e1000097. 10.1371/journal.pmed.1000097. 21603045 PMC3090117

[48] Gevorgyan H, Grigoryan GG, Atoyan HA, Rukhkyan M, Hakobyan A, Zakaryan H, et al. Evidence of Crimean-Congo haemorrhagic fever virus occurrence in Ixodidae ticks of Armenia. J Arthropod Borne Dis. 2019;13:9–16. 31346531 PMC6643020

[49] Hubálek Z, Sedláček P, Estrada-Peña A, Vojtíšek J, Rudolf I. First record of *Hyalomma rufipes* in the Czech Republic, with a review of relevant cases in other parts of Europe. Ticks Tick Borne Dis. 2020;11:101421. 10.1016/j.ttbdis.2020.101421. 32360146 10.1016/j.ttbdis.2020.101421

[50] Kampen H, Poltz W, Hartelt K, Wolfel R, Faulde M. Detection of a questing *Hyalomma marginatum marginatum* adult female (Acari, Ixodidae) in southern Germany. Exp Appl Acarol. 2007;43:227–31. 10.1007/s10493-007-9113-y. 17952610 10.1007/s10493-007-9113-y

[51] Weigand A, Teixeira J, Christian S. First record of *Hyalomma marginatum sensu stricto* C. L. Koch, 1844 and distribution of *Dermacentor reticulatus* (Fabricius, 1794) (Acari, Ixodidae) in Luxembourg. Bull Soc Nat Luxemb. 2020;122:253–63.

[52] Negredo A, Sánchez-Ledesma M, Llorente F, Pérez-Olmeda M, Belhassen-García M, González-Calle D, et al. Retrospective identification of early autochthonous case of Crimean-Congo hemorrhagic fever, Spain, 2013. Emerg Infect Dis. 2021;27:1754–6. 10.3201/eid2706.204643. 34013861 10.3201/eid2706.204643 PMC8153886

[53] Tonbak S, Aktas M, Altay K, Azkur AK, Kalkan A, Bolat Y, et al. Crimean-congo hemorrhagic fever virus: genetic analysis and tick survey in Turkey. J Clin Microbiol. 2006;44:4120–4. 10.1128/JCM.00644-06. 17088370 10.1128/JCM.00644-06 PMC1698322

[54] Braks MAH, van Wieren SE, Takken W, Sprong H, Jacobs F, Scholte EJ, et al. VectorNet: putting vectors on the map. Front Public Health. 2022;10:601371. 10.3389/fpubh.2022.601371. 10.3389/fpubh.2022.809763 PMC9013813 35444989

[55] Duscher GG, Hodžić A, Hufnagl P, Wille-Piazzai W, Schötta AM, Markowicz MA, et al. Adult *Hyalomma marginatum* tick positive for *Rickettsia aeschlimannii* in Austria, October 2018. Euro Surveill. 2018;23:1800595. 10.2807/1560-7917.ES.2018.23.48.1800595. 30621821 10.2807/1560-7917.ES.2018.23.48.1800595 PMC6280420

[56] European Centre for Disease Prevention and Control (ECDC). European network for medical and veterinary entomology (VectorNet). 2025. https://www.ecdc.europa.eu/en/about-us/partnerships-and-networks/disease-and-laboratory-networks/vector-net.

[57] Kaiser MN, Hoogstraal H, Watson GE. Ticks (Ixodoidea) on migrating birds in Cyprus, fall 1967 and spring 1968, and epidemiological considerations. Bull Entomol Res. 1974;34:97–110.

[58] Nuorteva P, Hoogstraal H. The incidence of ticks (Ixodoidea, Ixodidae) on migratory birds arriving in Finland during the spring of 1962. Ann Med Exp Biol Fenn. 1963;41:457–68. 14078774

[59] Walter C, Liebisch A, Vauk C. Untersuchungen zur biologie und verbreitung von Zecken (Ixodea, Ixodidae) in Norddeutschland. II. Zecken der Zugvögel auf der Insel Helgoland. Z Angew Zool. 1979;66:445–61.

[60] Kotlán S. What kinds of ixodids transmit piroplasmosis in Hungary? Allatorvosi Lapok. 1919;42:34–55. 10.5555/19211000356.

[61] Starkoff O. The ticks of Italy. Ixodea d’Italia. Rome: Il Pensiero Scientifico Editore; 1958.

[62] Pfliegler WP, Schönhofer A, Niedbała W, Vella P, Sciberras A, Vella A. New records of mites (Acari) and harvestmen (Opiliones) from Malta with a preliminary checklist of Maltese Arachnida. Soil Organisms. 2017;89:85–110.

[63] Nijhof AM, Bodaan C, Postigo M, Nieuwenhuijs H, Opsteegh M, Franssen L, et al. Ticks and associated pathogens collected from domestic animals in the Netherlands. Vector Borne Zoonotic Dis. 2007;7:585–95. 17979540 10.1089/vbz.2007.0130

[64] Mehl R, Michaelsen J, Lid G. Ticks (Acari, Ixodides) on migratory birds in Norway. Fauna Norvegica B. 1984;31:46–58.

[65] Cuber P. Ticks (Ixodida) from the collection of the Natural History Department, Museum of Upper Silesia in Bytom, Poland—a contribution to knowledge on tick fauna and the first record of *Hyalomma marginatum* presence in Poland. Ann Agric Environ Med. 2016;23:379–81. 10.5604/12321966.1203910. 27294652 10.5604/12321966.1203910

[66] Filipe AR, Casals J. Isolation of Dhori virus from *Hyalomma marginatum* ticks in Portugal. Intervirology. 1979;11:124–7. 10.1159/000149023. 85611 10.1159/000149023

[67] Nosek J, Kozuch O, Lysy J. The finding of the female *Hyalomma marginatum* Koch, 1844 in southern Slovakia. Folia Parasitol. 1982;29:251.

[68] Hoogstraal H, Kaiser MN, Taylor MA, Gaber S, Guindy E. Ticks (Ixodoidea) on birds migrating from Africa to Europe and Asia. Bull World Health Organ. 1961;24:197–212. 13715709 PMC2555510

[69] Parker RR, de Prada J, Bell EJ, Lackman DB. Recovery of *C. burnetii* from *H. savignyi* collected in Spain. Public Health Rep. 1949;64:1616–8. 10.2307/4587182. 15399325

[70] Brinck P, Svedmyr A, Zeipel G. Migrating birds at Ottenby, Sweden as carriers of ticks and possible transmitters of tick-borne encephalitis virus. Oikos. 1965;16:88–99. 10.2307/3564868.

[71] Aeschlimann A, Buttiker W. Importations of ticks into Switzerland (Acarina: Ixodea). Mitt Schweiz Entomol Ges. 1975;48:69–75.

[72] Göksu K. Systematic studies on theileria infection of cattle in Ankara. Vet Fak Yayml. Thesis. 1959;115:73.

[73] Wheler EG. British ticks. J Agric Sci. 1906;1:400–29. 10.1017/S0021859600000447.

[74] Hansford KM, Carter D, Gillingham EL, Hernandez-Triana LM, Chamberlain J, Cull B, et al. *Hyalomma rufipes* on an untraveled horse: is this the first evidence of *Hyalomma* nymphs successfully moulting in the United Kingdom? Ticks Tick Borne Dis. 2019;10:704–8. 10.1016/j.ttbdis.2019.03.003. 30876825 10.1016/j.ttbdis.2019.03.003

[75] Eltari E, Zeka S, Gina A, Sharofi F, Stamo K. Epidemiological data on some foci of haemorrhagic fever in our country. Rev Mjekesore. 1987;1:5–9.

[76] Matevosyan KSh, Semashko IV, Marutyan EM, Rubin SG, Chumakov MP. Discovery of Crimean haemorrhagic fever virus in the ticks *Hyalomma plumbeum plumbeum, Hyalomma anatolicum, Rhipicephalus bursa* and *Boophilus calcaratus* in the Armenian SSR. Tr Inst Poliomiel Virusn Entsef AMN SSSR. 1974;22:169–227.

[77] Hoogstraal H. The epidemiology of tick-borne Crimean-Congo hemorrhagic fever in Asia, Europe, and Africa. J Med Entomol. 1979;15:307–417. 113533 10.1093/jmedent/15.4.307

[78] Chinikar S, Ghiasi SM, Ghalyanchi-Langeroudi A, Goya MM, Shirzadi MR, Zeinali M, et al. An overview of Crimean-Congo hemorrhagic fever in Iran. Iran J Microbiol. 2009;1:7–12.

[79] Papa A, Christova I, Papadimitriou E, Antoniadis A. Crimean-Congo hemorrhagic fever in Bulgaria. Emerg Infect Dis. 2004;10:1465–7. 10.3201/eid1008.040162. 15496250 10.3201/eid1008.040162 PMC3320408

[80] Bernard C, Joly Kukla C, Rakotoarivony I, Duhayon M, Stachurski F, Huber K, et al. Detection of Crimean-Congo haemorrhagic fever virus in *Hyalomma marginatum* ticks, southern France, May 2022 and April 2023. Euro Surveill. 2024;29:2400023. 10.2807/1560-7917.ES.2024.29.6.2400023. 38333936 10.2807/1560-7917.ES.2024.29.6.2400023 PMC10853980

[81] Zakhashvili K, Tsertsvadze N, Chikviladze T, Jghenti E, Bekaia M, Kuchuloria T, et al. Crimean-Congo hemorrhagic fever in man, Republic of Georgia, 2009. Emerg Infect Dis. 2010;16:1326–8. 10.3201/eid1608.100097. 20678341 10.3201/eid1608.100181 PMC3298293

[82] Papadopoulos O, Koptopoulos G. Isolation of Crimean-Congo haemorrhagic fever (CCHF) virus from *Rhipicephalus bursa* ticks in Greece. Acta Microbiol Hell. 1978;23:20–8.

[83] Maltezou HC, Papa A, Tsiodras S, Dalla V, Maltezos E, Antoniadis A. Crimean-congo hemorrhagic fever in Greece: a public health perspective. Int J Infect Dis. 2009;13:713–6. 10.1016/j.ijid.2008.11.011. 19155182 10.1016/j.ijid.2008.11.011

[84] Antoniadis A, Casals J. Serological evidence of human infection with Congo-Crimean hemorrhagic fever virus in Greece. Am J Trop Med Hyg. 1982;31:1066–7. 10.4269/ajtmh.1982.31.1066. 6812443 10.4269/ajtmh.1982.31.1066

[85] Horváth LB. Precipitating antibodies to Crimean haemorrhagic fever virus in human sera collected in Hungary. Acta Microbiol Acad Sci Hung. 1976;23:331–5. 829015

[86] Mancuso E, Toma L, Polci A, d’Alessio SG, Di Luca M, Orsini M, et al. Crimean-Congo hemorrhagic fever virus genome in tick from migratory bird. Italy Emerg Infect Dis. 2019;25:1420. 10.3201/EID2507.181345. 10.3201/eid2507.181345 PMC6590740 31211933

[87] Fanelli A, Buonavoglia D, Lanave G, Monaco F, Quaranta V, Catanzariti R, et al. First serological evidence of Crimean-Congo haemorrhagic fever virus in transhumant bovines in Italy. Transbound Emerg Dis. 2022;69:4022–7. 10.1111/tbed.14710. 36150076 10.1111/tbed.14710 PMC10091806

[88] Chinikar S, Ghiasi SM, Hewson R, Moradi M, Haeri A. Crimean-congo hemorrhagic fever in Iran and neighbouring countries. J Clin Virol. 2010;47:110–4. 10.1016/j.jcv.2009.10.014. 20006541 10.1016/j.jcv.2009.10.014

[89] Filipe AR, Calisher CH, Lazuick J. Antibodies to Congo-Crimean haemorrhagic fever, Dhori, Thogoto and Bhanja viruses in southern Portugal. Acta Virol. 1985;29:324–8. 2864836

[90] Direção-Geral da Saúde. Crimean-Congo haemorrhagic fever case confirmed in Portugal [In Portuguese]. 2024. https://www.dgs.pt/em-destaque/confirmacao-de-caso-de-febre-hemorragica-de-crimeia-congo-em-portugal-.aspx.

[91] Ceianu CS, Panculescu-Gatej RI, Coudrier D, Bouloy M. First serologic evidence for the circulation of Crimean-Congo hemorrhagic fever virus in Romania. Vector Borne Zoonotic Dis. 2012;12:718–21. 10.1089/vbz.2011.0768. 22897346 10.1089/vbz.2011.0768

[92] European Centre for Disease Prevention and Control (ECDC). Crimean-Congo haemorrhagic fever: Prevention and control. 2008. https://www.ecdc.europa.eu/sites/default/files/media/en/publications/Publications/0809_MER_Crimean_Congo_Haemorragic_Fever_Prevention_and_Control.pdf.

[93] Karti SS, Odabasi Z, Korten V, Yilmaz M, Sonmez M, Caylan R, et al. Crimean-congo hemorrhagic fever in Turkey. Emerg Infect Dis. 2004;10:1379–84. 10.3201/eid1008.030928. 15496237 10.3201/eid1008.030928 PMC3320426

[94] Barr DA, Aitken C, Bell DJ, Brown CS, Cropley I, Dawood N, et al. First confirmed case of Crimean-Congo haemorrhagic fever in the UK. Lancet. 2013;382:1458. 10.1016/S0140-6736(13)61718-3. 24243135 10.1016/S0140-6736(13)61718-3

[95] Gligic A, Stamatovic L, Stojanovic R, Obradovic M, Boskovic R. The first isolation of the Crimean hemorrhagic fever virus in Yugoslavia. Vojnosanit Pregl. 1977;34:318–21. 414441

[96] Vesenjak-Hirjan J, Punda-Polić V, Dobe M. Geographical distribution of arboviruses in Yugoslavia. J Hyg Epidemiol Microbiol Immunol. 1991;35:129–40. 1658128

[97] Norman FF, Arce OA, Díaz-Menéndez M, Belhassen-García M, González-Sanz M. Changes in the epidemiology of Crimean-Congo hemorrhagic fever: impact of travel and a one health approach in the European region. Travel Med Infect Dis. 2025;64:102806. 10.1016/j.tmaid.2025.102806. 39870124 10.1016/j.tmaid.2025.102806

[98] Daněk O, Hrazdilová K, Kozderková D, Jirků D, Modrý D. The distribution of *Dermacentor reticulatus* in the Czech Republic re-assessed: citizen science approach to understanding the current distribution of the *Babesia canis* vector. Parasit Vectors. 2022;15:132. 10.1186/s13071-022-05242-6. 35436925 10.1186/s13071-022-05242-6 PMC9017003

[99] Földvári G, Szabó É, Tóth GE, Lanszki Z, Zana B, Varga Z, et al. Emergence of *Hyalomma marginatum* and *Hyalomma rufipes* adults revealed by citizen science tick monitoring in Hungary. Transbound Emerg Dis. 2022;69:e2240–8. 10.1111/tbed.14563. 35436033 10.1111/tbed.14563 PMC9790508

