# Supplementary figures and images for: Emergence and spread of Hyalomma ticks and Crimean-Congo haemorrhagic fever in Europe: a systematic review
Source: Parasit Vectors. 2025 Oct 28;18:436. doi: 10.1186/s13071-025-07104-3 (PMC12570448; doi:10.1186/s13071-025-07104-3)

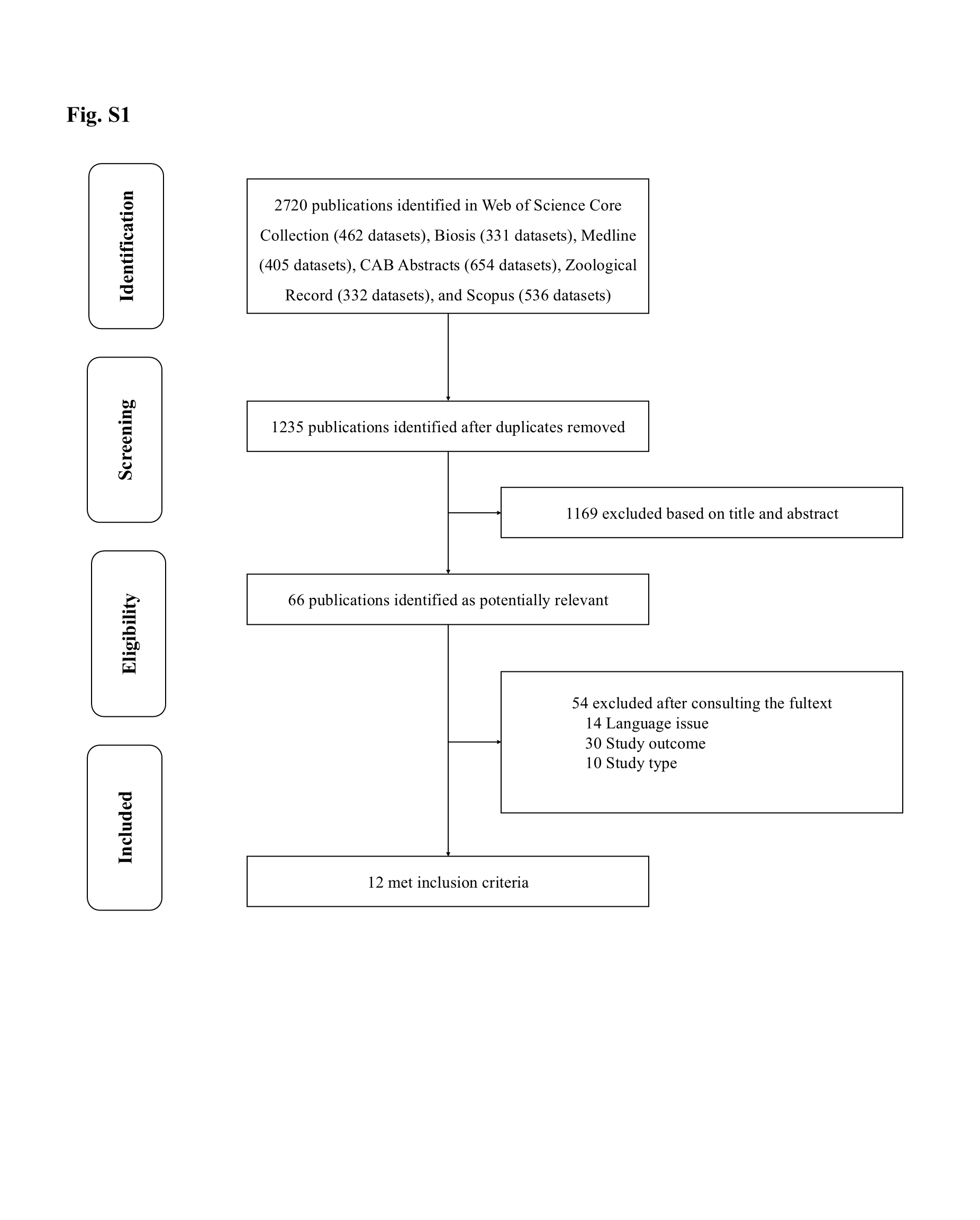

Supplement: Supplementary file 1 — Supplementary Material 1: Fig. S1. Preferred Reporting Items for Systematic Reviews and Meta-Analysesflow diagram illustrating the stages of the literature search process [file 13071_2025_7104_MOESM1_ESM.tif]
