# Supplementary material for: Emergence and spread of Hyalomma ticks and Crimean-Congo haemorrhagic fever in Europe: a systematic review
Source: Parasit Vectors. 2025 Oct 28;18:436. doi: 10.1186/s13071-025-07104-3 (PMC12570448; doi:10.1186/s13071-025-07104-3)
Supplement: Supplementary file 2 — Supplementary Material 2: Dataset S1. Articles retrieved through a systematic literature search that provided relevant information and references on Hyalomma, CCHF, or CCHFV for the target countries. [file 13071_2025_7104_MOESM2_ESM.docx]

**Supplementary Material**

List of articles that deemed directly relevant and provided references for the first detection of *Hyalomma* spp*.* / CCHF/ CCHFV:

1. Gevorgyan et al. (2019) Evidence of Crimean-Congo haemorrhagic fever virus occurrence in Ixodidae ticks of Armenia. J Arthropod Borne Dis. 13(1):9-16.
2. Grandi et al. (2020) First records of adult *Hyalomma marginatum* and *H. rufipes* ticks (Acari: Ixodidae) in Sweden. Ticks Tick Borne Dis. 11(3):101403. doi: https://doi.org/10.1016/j.ttbdis.2020.101403.
3. Hornok & Horváth (2012) First report of adult *Hyalomma marginatum rufipes* (vector of Crimean-Congo haemorrhagic fever virus) on cattle under a continental climate in Hungary. Parasit Vectors. 5:170. doi: <https://doi.org/10.1186/1756-3305-5-170>.
4. Hubálek et al. (2020) First record of *Hyalomma rufipes* in the Czech Republic, with a review of
   relevant cases in other parts of Europe. Ticks Tick Borne Dis. 11(4):101421. doi: https://doi.org/10.1016/j.ttbdis.2020.101421
5. Kampen et al. (2007) Detection of a questing *Hyalomma marginatum marginatum* adult female (Acari, Ixodidae) in southern Germany. Exp Appl Acarol 43, 227–231 (2007). <https://doi.org/10.1007/s10493-007-9113-y>
6. Negredo et al. (2021) Retrospective Identification of Early Autochthonous Case of Crimean-Congo Hemorrhagic Fever, Spain, 2013. Emerging Infectious Diseases 27 (6), pp. 1754-1756. DOI: 10.3201/eid2706.204643
7. Tonbak et al. (2006) Crimean-Congo hemorrhagic fever virus: Genetic analysis and tick survey in Turkey. J Clin Microbiol 44(11):4120-4. doi: 10.1128/JCM.00644-06. PMID: 17088370; PMCID: PMC1698322.
8. Weigand et al. (2020) First record of *Hyalomma marginatum* sensu strict C.L. Koch, 1844 and distribution of *Dermacentor reticulatus* (Fabricius, 1794) (Acari, Ixodidae) in Luxembourg. Bulletin de la Société des naturalistes luxembourgeois 122: 253-263.

List of articles that were not directly relevant but provided relevant references for the first detection of *Hyalomma* spp. / CCHF/ CCHFV

1. Bente et al. (2013) Crimean-Congo hemorrhagic fever: history, epidemiology, pathogenesis, clinical syndrome and genetic diversity. Antiviral Res. 100(1):159-89. doi: 10.1016/j.antiviral.2013.07.006.
2. Földvári et al. (2022) Emergence of *Hyalomma marginatum* and *Hyalomma rufipes* adults revealed by citizen science tick monitoring in Hungary. Transboundary and Emerging Diseases, 69(5), 2240-2248. **DOI:** 10.1111/tbed.14563
3. Hansford et al. (2019) *Hyalomma rufipes* on an untraveled horse: Is this the first evidence of *Hyalomma* nymphs successfully moulting in the United Kingdom? Ticks Tick Borne Dis. 10(3):704-708. doi: 10.1016/j.ttbdis.2019.03.003.
4. Jameson et al. (2012a) Importation of *Hyalomma marginatum*, vector of Crimean-Congo haemorrhagic fever virus, into the United Kingdom by migratory birds. Ticks Tick Borne Dis. 3(2):95-99. doi: 10.1016/j.ttbdis.2011.12.002.
